# Pyridachlometyl (Pesticides)

**DOI:** 10.14252/foodsafetyfscj.D-23-00002

**Published:** 2023-03-24

**Authors:** Food Safety Commission of Japan

## Abstract

Food Safety Commission of Japan (FSCJ) conducted a risk assessment of pyridachlometyl (CAS No.1358061-55-8), a pyridazine fungicide, based on results from various studies. The data used in the assessment include the fate in plants (wheat, sugar beet and others), residues in crops, fate in livestock (goats and chickens), residues in livestock, fate in animals (rats), and tests of subacute toxicity (rats, mice and dogs), chronic toxicity (dogs), combined chronic toxicity/carcinogenicity (rats), carcinogenicity (mice), two-generation reproductive toxicity (rats), developmental toxicity (rats and rabbits), genotoxicity and others. The major adverse effects of pyridachlometyl in experimental animals were observed in body weight (suppressed body weight gain), thyroid (increased weight, hypertrophy of follicular epithelial cell: rats and mice) and liver (increased weight, hepatocellar hypertrophy). No adverse effects were observed in the tests of fertility, teratogenicity or genotoxicity. The lowest no-observed-adverse-effect level (NOAEL) obtained from all the studies was 8 mg/kg bw per day in a two-year combined chronic toxicity/carcinogenicity study in rats. FSCJ specified an acceptable daily intake (ADI) of 0.08 mg/kg bw per day by applying a safety factor of 100 to the NOAEL. It is unnecessary to specify an acute reference dose (ARfD) because of adverse effects not expected to occur via a single administration of pyridacholometyl.

## Conclusion in Brief

Food Safety Commission of Japan (FSCJ) conducted a risk assessment of pyridachlometyl (CAS No.1358061-55-8), a pyridazine fungicide, based on results from various studies.

The data used in the assessment include the fate in plants (wheat, sugar beet and others), residues in crops, fate in livestock (goats and chickens), residues in livestock, fate in animals (rats), and tests of subacute toxicity (rats, mice and dogs), chronic toxicity (dogs), combined chronic toxicity/carcinogenicity (rats), carcinogenicity (mice), two-generation reproductive toxicity (rats), developmental toxicity (rats and rabbits), genotoxicity and others.

The major adverse effects of pyridachlometyl in experimental animals were observed in body weight (suppressed body weight gain), thyroid (increased weight, hypertrophy of follicular epithelial cell: rats and mice) and liver (increased weight, hepatocellar hypertrophy). No adverse effects were observed in the tests of fertility, teratogenicity or genotoxicity.

Following incidences were observed.

∙ Thyroid follicular cell adenomas/carcinomas, hepatocellular adenomas/carcinomas and endometrial stromal polyp in a two-year combined chronic toxicity/carcinogenicity study in rats.

∙ Hepatocellular adenomas/carcinomas in an eighteen-month carcinogenicity study in mice.

These tumors are likely to occur through non-genotoxic mechanisms because of the result of genotoxicity tests. The judgement allowed to establish a threshold value of this chemical. The endometrial stromal polyp has different morphological and pathological characteristics between rodents and humans, it is thus concluded that the relevance and extrapolation to a corresponding human tumor are low. In addition, mechanistic studies indicates the tumors in hepatocytes and thyroid follicular cells in rodents for none or low extrapolation values to humans.

On the basis of the results of various studies, the only parent compound was identified as the substance relevant to the residue definition for the dietary risk assessment in agricultural products and livestock products of pyridachlometyl.

The lowest no-observed-adverse-effect level (NOAEL) obtained from all the studies was 8 mg/kg bw per day in a two-year combined chronic toxicity/carcinogenicity study in rats. FSCJ specified an acceptable daily intake (ADI) of 0.08 mg/kg bw per day by applying a safety factor of 100 to the NOAEL.

It is unnecessary to specify an acute reference dose (ARfD) because of adverse effects not expected to occur via a single administration of pyridacholometyl.

**Table 1.** *Levels relevant to toxicological evaluation of pyridachlometyl*

Species	Study	Dose (mg/kg bw per day)	NOAEL (mg/kg bw per day)	LOAEL (mg/kg bw per day)	Critical endpoints^1^^)^
Rat	90-day subacute neurotoxicity study (the 1^st^ study)	0, 1 000, 5 000, 20 000 ppm	M: 56 F:72	M: 291 F: 351	M/F: Thyroid follicular epithelial cell hypertrophy, centrilobular hepatocellular hypertrophy and others
		M: 0, 56, 291, 1 190 F: 0, 72, 351, 1 330			
	90-day subacute neurotoxicity study (the 2^nd^ study)	0, 2000, 5 000, 10 000, 20 000 ppm	M: - F: 136	M: 117 F: 337	M/F: Hepatocellular hypertrophy, thyroid follicular epithelial cell hypertrophy and others
		M: 0, 117, 312, 621, 1 230 F: 0, 136, 337, 665, 1 440			
	90-day subacute neurotoxicity study FSCJ’s general aspect from (the 1^st^ study) and (the 2^nd^ study)^2)^		M: 56 F: 72		
	Two-year combined chronic toxicity/ carcinogenicity study	0, 200, 2 000, 10 000, 20 000 ppm	M: 8 F: 10	M: 87 F: 99	M: Increase of GGT, panlobular hepatocellular hypertrophy and others F: Centrilobular hepatocllular hypertrophy, thyroid colloidal degeneration and others (M/F: Increased incidences of thyroid follicular cell adenomas and follicular cell carcinomas F: Increased incidences of hepatocellular adenomas/ hepatocellular carcinomas and endometrial stromal polyp)
		Carcinogenicity study group M: 0, 8, 87, 451, 916 F: 0, 10, 99, 516, 1 060 52 week emergently slaughtered group M: 0, 9, 92, 488, 976 F: 0, 11, 111, 576, 1 190			
	Two-generation reproductive toxicity study	0, 600, 4 000, 20 000 ppm	Parent: PM: 36 PF: 39 F_1_M: 46 F_1_F: 47 Offspring: PM: 242 PF: 257 F_1_M: 311 F_1_F: 321	Parent: PM: 242 PF: 257 F_1_M: 311 F_1_F: 321 Offspring: PM: 1 270 PF: 1 320 F_1_M: 1 670 F_1_F: 1 730	Parent: M/F: Thyroid follicular epithelial cell hypertrophy and others Offspring: M/F: Suppressed body weight gain (No effect on fertility is observed)
		PM: 0, 36, 242, 1 270 PF: 0, 39, 257, 1 320 F_1_M: 0, 46, 311, 1 670 F_1_F: 0, 47, 321, 1 730			
	Developmental toxicity study	0, 250, 500, 1 000	Dams: 1 000 Fetuses: 1 000	Dams: - Fetuses: -	Dams: No toxicity is observed. Fetuses: No toxicity is observed. (No teratogenicity is observed)
Mouse	90-day subacute toxicity study	0, 1 500, 3 500, 7 000 ppm	M: 216 F: 650	M: 517 F: 1 150	M: Hepatocellular hypertrophy and thyroid follicular epithelial cell hypertrophy F: Increased T. Chol, hepatocellular hypertrophy, thyroid follicular epithelial cell hypertrophy and others
		M: 0, 216, 517, 995 F: 0, 252, 650, 1 150			
	18-month carcinogenicity study	0, 700, 2 000, 7 000 ppm	M: 83 F: 114	M: 242 F: 317	M/F: Increased absolute and relative organ weights of liver, liver cell hypertrophy and others (M: Increased incidences of hepatocellular adenomas/hepatocellular carcinomas)
		Carcinogenicity study group M: 0, 83, 242, 848 F: 0, 114, 317, 1 110 52 week emergently slaughtered group M: 0, 84, 238, 866 F: 0, 122, 299, 1 070			
Rabbit	Developmental toxicity study	0, 250, 500, 1 000	Dams: 250 Fetuses: 1 000	Dams: 500 Fetuses: -	Dams: Decrease of faces and food intake Fetuses: No toxicity is observed. (No teratogenicity is observed)
Dog	90-day subacute neurotoxicity study	0, 100, 300, 1000	M/F: -	M/F: 100	M/F: Increased GGT, centrilobular hepatocellular hypertrophy and others
	One-year chronic toxicity study	0, 10, 50, 300	M/F: 10	M/F: 50	M/F: Increased ALT and ALP, centrilobular hepatocellular hypertrophy and others
ADI			NOAEL: 8 SF: 100 ADI: 0.08		
The critical study for setting ADI			Two-year combined chronic/carcinogenicity study (rat)		

ADI, Acceptable daily intake; NOAEL, No-observed-adverse-effect level; SF, Safety factor; -, NOAEL or LOAEL could not be specified; /, Not applicable

^1)^ The adverse effect observed at LOAEL

^2)^ The FSCJ takes conservative aspect to identify NOAEL from the two tests (the 1^st^ study) and (the 2^nd^ study).
